# *Serratia* Causing Liver Abscess in a Young Immunocompetent Female: A Rare Case With Literature Review

**DOI:** 10.1155/crdi/7239044

**Published:** 2026-10-21

**Authors:** Malak R. Hroub, Mohammed A. Barakat, Alhareth M. Amro, Mohanad A. Abuzahra, Maha Ramzi, Omar H. Salloum, Basheer Babaa

**Affiliations:** ^1^ Faculty of Medicine Al-Quds University Jerusalem West Bank State of Palestine alquds.edu; ^2^ Department of Radiology Rafidia Hospital Nablus West Bank State of Palestine; ^3^ Department of Radiology Istishari Arab Hospital Ramallah West Bank State of Palestine; ^4^ Faculty of Health Professions Al-Quds University Jerusalem West Bank State of Palestine alquds.edu; ^5^ Faculty of Medicine Palestine Polytechnic University Hebron West Bank State of Palestine ppu.edu

**Keywords:** abdominal CT, fever of unknown origin, immunocompetent host, multidisciplinary management, pyogenic liver abscess, rare case report, *Serratia marcescens*

## Abstract

**Background:**

Pyogenic liver abscess (PLA) is a life‐threatening condition predominantly caused by enteric Gram‐negative bacilli in patients with diabetes, hepatobiliary disease, or immunosuppression. *Serratia marcescens* is a well‐known opportunistic pathogen, typically causing nosocomial infections in compromised hosts. Community‐acquired PLA due to *S. marcescens* in healthy individuals is exceedingly rare.

**Case Presentation:**

A 28‐year‐old immunocompetent postpartum female presented with a 40‐day history of low‐grade fever following a cesarean section, accompanied by fatigue, lower abdominal discomfort, and recent right upper quadrant pain. Initial laboratory investigations revealed marked leukocytosis, microcytic anemia, thrombocytosis, and elevated C‐reactive protein (318.76 mg/L). Abdominal computed tomography (CT) demonstrated an 8.5 × 5.8 cm multiloculated hypodense hepatic abscess in segment IV. CT‐guided needle aspiration yielded purulent material; pus cultures selectively grew *S. marcescens*. Concurrently, extended‐spectrum beta‐lactamase (ESBL)–producing *Escherichia coli* and *Enterococcus faecalis* were isolated from urine, while *Staphylococcus haemolyticus* grew in a single blood culture bottle. Target antimicrobial therapy was adjusted to intravenous meropenem, leading to rapid clinical and inflammatory marker improvement. Because the patient demonstrated prompt clinical stabilization following diagnostic aspiration, percutaneous catheter placement was deferred by a multidisciplinary team. She completed a course of IV meropenem followed by 4 weeks of oral trimethoprim–sulfamethoxazole. Follow‐up ultrasound at 1 month confirmed clinical resolution and regression of the abscess cavity to 1.0 cm.

**Conclusion:**

*S. marcescens* should be considered in the differential diagnosis of PLA, even in immunocompetent individuals presenting with prolonged fever of unknown origin. Early cross‐sectional imaging, microbiological aspiration, and multidisciplinary care are essential for prompt resolution without invasive surgical drainage.

## 1. Background

Pyogenic liver abscess (PLA) represents a localized collection of purulent exudate within the hepatic parenchyma arising from direct contiguous spread, portal venous drainage, or systemic bacteremia [1]. The bulk of these abscesses are classified as pyogenic or amoebic, with a few caused by parasites and fungi [2]. Globally, the incidence of PLA demonstrates regional variability, ranging from 1.1 to 3.6 per 100,000 person‐years in Western nations to upwards of 17.6 per 100,000 in East Asia [3]. Liver abscesses may be solitary or multiple. Biliary abscesses are typically multiple and impact both liver lobes, whereas lesions that extend through the portal vein, through continuity, or as a result of trauma are single. They are more commonly found in the right hepatic lobe than in both or left hepatic lobes (60%–70% vs. 25% and 15% cases, respectively) [1].

*Serratia* species, notably *Serratia marcescens*, are known as opportunistic pathogens that can cause a variety of illnesses, including pneumonia, urinary tract infections, and, less frequently, liver abscesses [4]. Predisposing risk factors for *Serratia* acquisition typically include underlying malignancy, poorly controlled diabetes mellitus, alcohol dependency, severe thermal injury, or primary immunodeficiencies such as chronic granulomatous disease [5]. Despite the known occurrences of liver abscesses caused by several *Serratia* species, reports specifically involving these infections in immunocompetent individuals remain rare.

Development of a community‐acquired PLA caused by *S. marcescens* in an immunocompetent patient without traditional risk factors is an extraordinarily rare clinical event. Herein, we describe the clinical course, diagnostic workup, and successful nonsurgical multidisciplinary management of an 8.5‐cm *S. marcescens* PLA in a 28‐year‐old postpartum female, supplemented by a focused literature review.

## 2. Case Presentation

### 2.1. Patient History and Timeline

A 28‐year‐old female (Gravida 2, Para 2) presented to the medical center for evaluation of a prolonged, insidious illness spanning 40 days. Her symptoms began on postpartum day 2 following an uncomplicated repeat elective cesarean section (CS) performed 6 weeks prior. The illness began with low‐grade intermittent fevers (38.0°C–39.0°C) lasting 1 week, partially responsive to oral paracetamol. Over the subsequent 4 weeks, she suffered progressive fatigue, leaving her largely bedridden, accompanied by lower back pain, pelvic discomfort, foul‐smelling vaginal discharge without pruritus, and irregular post‐CS vaginal spotting. Notably, she experienced an intentional prepregnancy/gestational weight reduction from 110 to 80 kg. During the 5th week of illness, her symptoms localized to a vague right upper quadrant (RUQ) abdominal ache.

Her past medical history was significant for a left salpingectomy in 2022 and a laparoscopic cholecystectomy. She had no known chronic illnesses, drug allergies, or history of anticoagulant use. Her menstrual cycles were irregular, with the last menstrual period recorded on October 20, 2024. She was lactating at the time of presentation.

### 2.2. Diagnostic Assessment and Therapeutic Interventions

Upon admission, her vital signs were stable (BP 120/70 mmHg, temperature 36.2°C), and she was alert and oriented (GCS: 15/15). Initial examination revealed mild cervical motion tenderness on pelvic examination but no notable vaginal discharge. Pelvic ultrasound showed an empty uterus with no adnexal mass.

Initial laboratory evaluation revealed marked acute systemic inflammation and microcytic anemia:

• Complete blood count: White blood cell (WBC) count 11.4 × 10⁹/L (neutrophils 78%, left‐shift present), hemoglobin 7.4 g/dL, hematocrit 24.1%, and platelet count 810 × 10^9^/L.
• Inflammatory markers: C‐reactive protein (CRP) 318.76 mg/L (normal < 5 mg/L) and erythrocyte sedimentation rate (ESR) 115 mm/h.
• Liver function tests: total bilirubin 0.8 mg/dL, direct bilirubin 0.3 mg/dL, alanine aminotransferase (ALT) 42 U/L, aspartate aminotransferase (AST) 38 U/L, alkaline phosphatase (ALP) 210 U/L (mild elevation), gamma‐glutamyl gransferase (GGT) 85 U/L, and serum albumin 2.8 g/dL.
• Metabolic and serology: serum potassium 5.13 mmol/L and serum sodium 138 mmol/L.

Blood smear was negative for lymphoproliferative blastic cells. Serological testing for HIV, HBV, HCV, *Brucella abortus/melitensis*, and the Widal test were all negative. The autoimmune panel (ANA, ANCA, and anti‐dsDNA) was unrevealing.

### 2.3. Radiological Evaluation and Microbiological Workup

Pelvic ultrasound showed a normal involuted empty uterus with normal bilateral adnexa. Initial abdominal ultrasound was largely unremarkable except for a suspected 1.5‐cm hepatic hemangioma in the right lobe. Due to persistent fever, sweating, and weight loss with negative cultures, a whole‐body computed tomography (CT) scan was performed on hospital day 3, revealing a large hypodense hepatic lesion in Segment IV (8.5 × 5.8 cm), with heterogeneous enhancement and irregular margins, favoring a hepatic abscess (Figure 1).

**FIGURE 1:**
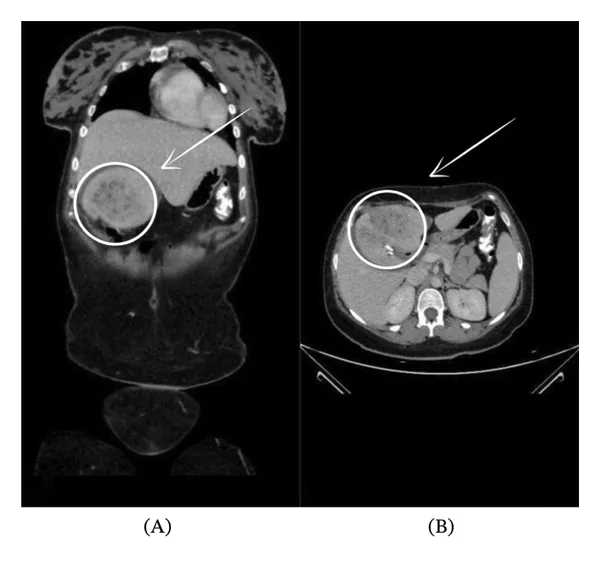
Initial contrast‐enhanced abdominal CT scan (axial view) demonstrating a large, multiloculated hypodense hepatic abscess measuring 8.5 × 5.8 cm in Segment IV (arrow), featuring peripheral rim enhancement and surrounding parenchymal edema. Coronal (A) and axial (B) contrast‐enhanced CT images of the liver in the portal venous phase reveal a large hypodense mass centered in Segment IV. The lesion exhibits heterogeneous and irregular enhancement, with irregular septations and interspersed nonenhancing areas. Its margins are ill‐defined and irregular.

Image‐guided percutaneous needle aspiration (PNA) yielded 30 mL of frank purulent material. Microbiological culture of the aspirated pus isolated pure growth of *S. marcescens*, sensitive to meropenem, ciprofloxacin, and trimethoprim–sulfamethoxazole but resistant to ampicillin, cefazolin, and cefuroxime.

Concurrent microbiological investigations demonstrated the following:

1. Urine culture and vaginal swab: isolated extended‐spectrum beta‐lactamase (ESBL)–producing *Escherichia coli* (> 10^5^ CFU/mL) and *Enterococcus faecalis.*
2. Blood culture: 1 of 2 bottle sets grew *Staphylococcus haemolyticus* (interpreted as a skin contaminant/commensal given clinical stability and rapid clearance).

The diagnosis of the persistent fever of unknown origin was attributed to the hepatic abscess (*Serratia* spp.) with coincidence of urinary tract infection (ESBL *E. coli* and *Enterococcus faecalis*).

### 2.4. Therapeutic Interventions and Clinical Decision‐Making

Initial empirical antimicrobial coverage with IV ceftriaxone and IV clindamycin was commenced. Upon identification of *S. marcescens* from abscess fluid and ESBL‐producing *E. coli* in the urinary tract, antibiotic treatment was escalated to IV meropenem.

A multidisciplinary team (MDT) including general surgery, interventional radiology, and infectious disease specialists was consulted. The general surgery team deemed surgery unnecessary, while interventional radiology advised against reaspiration due to the small abscess size. The infectious disease team recommended continuing IV meropenem for another week.

By the time of discharge, the patient was afebrile, ambulatory, and clinically improved. She was discharged on oral sulfamethoxazole–trimethoprim (Sulprim Forte) for 4 weeks with a follow‐up abdominal CT scan scheduled after 1 month.

### 2.5. Follow‐Up and Outcomes

Internal medicine follow‐up was arranged for 1 week postdischarge. Follow‐up abdominal ultrasound showed a decrease in abscess size to approximately 1 cm. Clinical evaluation demonstrated significant improvement, with almost complete resolution of the hepatic abscess (Figure 2) (see Table 1).

**FIGURE 2:**
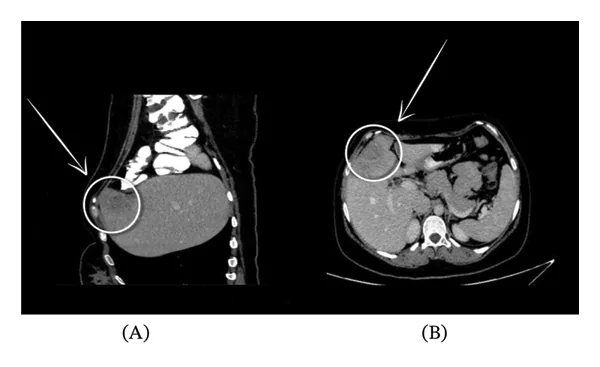
Follow‐up abdominal CT scan (axial view) obtained 4 weeks posttreatment demonstrating near‐complete resolution of the Segment IV hepatic abscess, leaving a small residual fibrotic scar/cavity measuring approximately 1.0 cm. Sagittal (A) and axial (B) CT images obtained in the portal venous phase 1 month after treatment show a hypodense region at the site of the known abscess, decreased in size, suggesting improvement.

**TABLE 1:** CARE guideline clinical timeline.

Timepoint	Clinical events/findings	Management/outcome
Day −40 to Day 0	Elective CS performed; onset of intermittent fever, fatigue, pelvic pain, and progressive RUQ ache	—
Day 1 (admission)	Hospitalization; baseline labs: CRP 318 mg/L, Hb 7.4 g/dL	Empirical IV ceftriaxone + clindamycin initiated
Day 3	Contrast CT identifies 8.5 × 5.8 cm Segment IV liver abscess	CT‐guided needle aspiration performed
Day 5	Pus culture confirms *Serratia marcescens*; urine/vaginal swab confirms ESBL *E. coli*	MDT consultation; regimen changed to IV meropenem 1 g q8h; PCD deferred
Day 12	Complete defervescence; CRP decreased to 16.0 mg/L	Discharged on oral trimethoprim–sulfamethoxazole (Sulprim Forte) 800/160 mg BID
Day 40 (1‐month postdischarge)	Follow‐up CT shows lesion shrinkage to ∼1.0 cm; full symptomatic recovery	Continued clinical recovery; marked radiological resolution

## 3. Discussion

PLAs present a significant diagnostic and therapeutic challenge due to their often‐subtle clinical manifestations and potential for serious complications if left untreated. The majority of cases are attributed to enteric Gram‐negative bacilli, most commonly *Klebsiella pneumoniae* and *E. coli*, with anaerobic organisms frequently contributing to polymicrobial infections [6]. These infections are typically seen in individuals with well‐recognized risk factors such as diabetes mellitus, underlying hepatobiliary disease, immunosuppression, or a history of recent abdominal surgery [7]. Against this backdrop, the present case is particularly noteworthy, as it involves a rare causative organism—*Serratia* species—identified in an immunocompetent young woman without any of the conventional predisposing factors. The patient, a postpartum female, had no prior history of diabetes, malignancy, or immunosuppressive conditions, making this an unusual and clinically significant presentation.

*S. marcescens* is a facultative anaerobic, Gram‐negative bacillus from the Enterobacteriaceae family [8]. It is well recognized for causing nosocomial infections such as catheter‐associated urinary tract infections, pneumonia, and bacteremia, especially in immunocompromised patients or those who have been in the hospital for an extended period of time and have undergone invasive operations. *Serratia*’s appearance as a pathogen in community‐acquired PLA is highly unusual, especially in the absence of conventional predisposing conditions [9].

PLAs are often characterized by nonspecific symptoms such as fever, RUQ pain, lethargy, and weight loss. In rare situations, especially early in the disease course or in unusual presentations, symptoms can be vague and misleading, mimicking other abdominal or systemic illnesses [10]. The underlying etiology, patient comorbidities, and the virulence of the causative organism can all have an impact on the clinical presentation. Immunocompromised individuals or those with hepatobiliary illness may appear more intensely, but otherwise healthy persons may proceed more slowly, resulting to diagnostic delays [11]. In our case, the patient was a 28‐year‐old immunocompetent postpartum female who presented with a prolonged history of intermittent fever that began shortly after cesarean delivery. Associated symptoms included progressive fatigue, lower abdominal and back pain, weight loss, menstrual irregularities, and foul‐smelling vaginal discharge, features that initially suggested a genitourinary source of infection. Despite empirical treatment, her fever persisted, and her condition gradually deteriorated over several weeks, making the clinical presentation more complex. The subtlety and duration of her symptoms in the absence of classical risk factors for liver abscess obscured the initial diagnostic impression and warranted further investigation.

The diagnosis of PLA involves a combination of clinical suspicion, laboratory findings, and radiological imaging, with confirmation through microbiological identification of the causative organism [12]. Elevated inflammatory markers, such as leukocytosis and CRP, often serve as initial indicators of infection. While abdominal ultrasonography is commonly the first‐line imaging modality, its sensitivity may be limited in early or atypical cases. Contrast‐enhanced CT offers greater diagnostic accuracy and is often employed when clinical suspicion remains high despite inconclusive initial tests. Definitive diagnosis is achieved through image‐guided aspiration of the lesion, allowing for microbial culture and targeted therapy [13]. In this case, the patient’s persistently elevated CRP (318 mg/L), anemia, thrombocytosis, and leukocytosis raised concern for a deep‐seated infection. Initial ultrasound imaging was nondiagnostic, prompting a CT scan, which revealed a large hypodense lesion in Segment IV of the liver with irregular margins, suggestive of a hepatic abscess. Image‐guided aspiration confirmed the presence of pus, and microbiological analysis identified *Serratia* species as the etiological agent, an uncommon finding in immunocompetent individuals. This confirmed the diagnosis of a PLA due to *Serratia*, guiding appropriate and timely antimicrobial management.

Our patient’s treatment followed established principles for PLA, initiating with empirical broad‐spectrum parenteral antibiotic therapy (ceftriaxone and clindamycin), consistent with recommended first‐line approaches [1]. Identification of *Serratia* spp. from the abscess aspirate, along with ESBL‐producing organisms from other sites, prompted a switch to meropenem to ensure adequate coverage against identified pathogens and potential resistance, following the recommendations to modify therapy based on pus/blood culture results for such cases [14]. CT‐guided PNA was performed initially, primarily for microbiological examination to guide targeted therapy. Although percutaneous catheter drainage (PCD) or surgical drainage is often indicated for larger abscesses (> 5 cm) or complex cases [15], the MDT determined that further drainage was unnecessary in this case due to significant clinical improvement and abscess size reduction with medical management following the initial aspiration. The total duration of treatment involved extended intravenous administration followed by oral therapy, aligning with the recommended minimum treatment duration of 2–6 weeks [1], ultimately resulting in nearly complete resolution.

Table 2 illustrates that *Serratia*‐related liver abscesses are predominantly reported in immunocompromised individuals, often with underlying conditions such as diabetes, malignancy, or chronic granulomatous disease. Most cases required drainage and extended antibiotic therapy. To date, only two cases—including the current report—have documented *Serratia* liver abscesses in immunocompetent adults. Notably, the case reported by Balakrishna et al. involved *Serratia grimesii* [18], making our case the first documented instance of a *S. marcescens* PLA in an immunocompetent postpartum female. This underscores the importance of considering atypical pathogens like *S. marcescens* even in healthy individuals, especially when standard treatments fail or fever persists.

**TABLE 2:** Reported cases of *Serratia*‐associated liver abscesses in the literature.

First author	Year	Age/sex	Pathogen	Underlying conditions	Therapy	Suspected cause
Our case	2024	28/F	*Serratia marcescens*	Free past medical history, no recent surgeries.	Drainage + IV meropenem for 2 weeks then by sulfamethoxazole–trimethoprim for 4 weeks	Unknown

Perez [16]	2023	32/M	*Serratia marcescens*	Not described East Asian Cruise line worker	Drainage + IV metronidazole and ceftriaxone	Not speculated

Fujikawa and Araki [17]	2020	86/F	*Serratia marcescens*	Dementia, osteoporosis, elderly	Not described	Not speculated

Erem et al. [5]	2019	45/F	*Serratia marcescens* *Streptococcus viridans*	Diabetes, severe heart failure, microcytic anemia, immunocompromised	Drainage + IV ceftriaxone and oral metronidazole	Immunocompromised, diabetes

Balakrishna [18]	2018	42/M	*Serratia grimesii*	Immunocompetent (no details provided)	Piperacillin and tazobactam for 11 days	Not speculated

Khattou et al. [19]	2018	2/F	*Serratia marcescens*	Chronic granulomatous disease (immunocompromised)	Ceftriaxone + gentamicin + metronidazole (not more details)	Chronic granulomatous disease (immunocompromised)

Hashemi [20]	2016	59/M	*Serratia ficaria*	Cholecystitis Fig trees trimming	Ciprofloxacin and ertapenem for several weeks	Infection from trimming a fig tree and recent surgery

Watanabe et al. [21]	2005	69/F	*Serratia marcescens*	Pancreatic cancer (immunocompromised)	Ciprofloxacin for 13 days	Suspected to be from drainage tube placement

Herman and Siegel [22]	2002	1 week/M	*Serratia marcescens*	Chronic granulomatous disease (immunocompromised)	Not described	Chronic granulomatous disease (immunocompromised)

Grieco et al. [23]	1973	32/M	*Serratia marcescens*	Alcoholism, liver disease, melena (immunocompromised)	Gentamicin and polymyxin for 6 & 3 months	Multiple surgeries

*Note:* F: female, M: male.

Although the patient was classified as immunocompetent, it is worth noting that the postpartum period can be associated with transient immune modulation and changes in mucosal barriers [24]. We hypothesize that a mild ascending postpartum infection after cesarean delivery, supported by foul‐smelling discharge and ESBL *E. coli* colonization, may have facilitated the spread of opportunistic pathogens to the liver. Alternatively, the prior laparoscopic cholecystectomy in 2022 could have altered local biliary dynamics.

Complications of liver abscesses can include sepsis, rupture into adjacent structures, peritonitis, and chronic residual cavities [25]. Fortunately, our patient did not exhibit signs of systemic sepsis or abscess rupture. Follow‐up imaging 1 month after discharge demonstrated a significant reduction in abscess size to approximately 1 cm, correlating with marked clinical improvement and normalization of inflammatory markers. Continued oral antibiotic therapy and close outpatient follow‐up ensured complete resolution without recurrence or long‐term complications. This highlights the importance of sustained monitoring postdischarge to detect potential relapse or treatment failure in similar cases.

For large PLA (> 5 cm), guidelines generally favor catheter drainage (PCD) over needle aspiration (PNA) due to lower failure rates. However, recent evidence [26] suggests that PNA with potent bactericidal therapy may be effective in selected patients. In our patient, rapid improvement after PNA and targeted meropenem eliminated the need for catheterization, reducing hospital stay and catheter‐related morbidity. The single‐bottle *S. haemolyticus* isolate was considered a contaminant, while resolution of the ESBL *E. coli* UTI further supported meropenem’s efficacy.

This case expands the current understanding of PLA by illustrating that even in the absence of conventional risk factors, rare and opportunistic pathogens like *S. marcescens* should be considered in patients with prolonged fever and systemic symptoms, particularly in the postpartum period. Early imaging, microbiological confirmation, and multidisciplinary care remain key pillars in ensuring accurate diagnosis and favorable outcomes.

## 4. Conclusion

This case highlights an exceptionally rare presentation of a PLA caused by *S. marcescens* in an immunocompetent, postpartum young female—an organism more commonly associated with nosocomial infections in immunocompromised individuals. The subtle and prolonged symptomatology, coupled with an atypical causative agent, posed a significant diagnostic challenge. Early suspicion, comprehensive imaging, and microbiological confirmation were pivotal to accurate diagnosis and effective treatment. Clinicians should maintain a broad differential when evaluating fever of unknown origin, even in immunocompetent hosts, and remain vigilant for uncommon pathogens, particularly in the postpartum period. This case underscores the importance of multidisciplinary management and tailored antimicrobial therapy in achieving successful outcomes.

**Nomenclature**
PLA: Pyogenic liver abscess
CT: Computed tomography
CS: Cesarean section
ESBL: Extended‐spectrum beta‐lactamase
CRP: C‐reactive protein
CBC: Complete blood count
Hb: Hemoglobin
PLTs: Platelets
WBCs: White blood cells
HIV: Human immunodeficiency virus
HCV: Hepatitis C virus
HBV: Hepatitis B virus
MDT: Multidisciplinary team
PNA: Percutaneous needle aspiration
PCD: Percutaneous catheter drainage
GCS: Glasgow coma scale

## Author Contributions

Malak R. Hroub, Mohammed A. Barakat, and Alhareth M. Amro contributed equally to the conception, data collection, literature review, drafting of the manuscript, and critical intellectual input.

Mohanad A. Abuzahra, Omar H. Salloum, Maha Ramzi, and Basheer Babaa were actively involved in the conception of the clinical approach, interpretation of clinical findings, preparation of imaging and radiological descriptions, and provided critical revision of the manuscript. They also played a key role in refining the discussion and ensuring the accuracy of the clinical context and implications.

All authors made substantial contributions to the conception of the work, acquisition, and were involved in drafting or critically revising the manuscript;

## Funding

The authors have not received any payments or services, either directly or indirectly from a third party in support of any aspect of this work.

## Disclosure

All authors provided final approval of the version to be published and agreed to be accountable for all aspects of the work in ensuring the integrity and accuracy of its content.

## Ethics Statement

The authors have nothing to report.

## Consent

Informed consent to publish this case report was provided by the patient.

## Conflicts of Interest

The authors declare no conflicts of interest.

## Supporting Information

Additional supporting information can be found online in the Supporting Information section.

## Supporting information


**Supporting Information** CARE checklist.

## Data Availability

No datasets were generated or analyzed during the current study.
