# Supplementary material for: Serratia Causing Liver Abscess in a Young Immunocompetent Female: A Rare Case With Literature Review
Source: Case Rep Infect Dis. 2026 Oct 21;2026:7239044. doi: 10.1155/crdi/7239044 (PMC13687591; doi:10.1155/crdi/7239044)
Supplement: Supplementary file 1 [file CRDI-2026-7239044-s001.pdf]

CARE case report guidelines

CARE Checklist of information to include when writing a case report

(cc) BY-NC-ND

Topic	Item	Checklist item description	Reported on Line
Title	1	The diagnosis or intervention of primary focus followed by the words "case report"	2
Key Words	2	2 to 5 key words that identify diagnoses or interventions in this case report, including "case report"	54
Abstract (no references)	3a	Introduction: What is unique about this case and what does it add to the scientific literature?	48-51
	3b	Main symptoms and/or important clinical findings	44
	3c	The main diagnoses, therapeutic interventions, and outcomes	44-47
	3d	Conclusion—What is the main "take-away" lesson(s) from this case?	50-51
Introduction	4	One or two paragraphs summarizing why this case is unique (**may include references**)	80-92
Patient Information	5a	De-identified patient specific information.	94
	5b	Primary concerns and symptoms of the patient.	94
	5c	Medical, family, and psycho-social history including relevant genetic information	104-106
	5d	Relevant past interventions with outcomes	103
Clinical Findings	6	Describe significant physical examination (PE) and important clinical findings.	110-113
Timeline	7	Historical and current information from this episode of care organized as a timeline	110-120
Diagnostic Assessment	8a	Diagnostic testing (such as PE, laboratory testing, imaging, surveys).	118-131
	8b	Diagnostic challenges (such as access to testing, financial, or cultural)	132-137
	8c	Diagnosis (including other diagnoses considered)	132-135
	8d	Prognosis (such as staging in oncology) where applicable	
Therapeutic Intervention	9a	Types of therapeutic intervention (such as pharmacologic, surgical, preventive, self-care)	124, 137
	9b	Administration of therapeutic intervention (such as dosage, strength, duration)	137,139
	9c	Changes in therapeutic intervention (with rationale)	135-136
Follow-up and Outcomes	10a	Clinician and patient-assessed outcomes (if available)	138
	10b	Important follow-up diagnostic and other test results	142-143
	10c	Intervention adherence and tolerability (How was this assessed?)	
	10d	Adverse and unanticipated events	
Discussion	11a	A scientific discussion of the strengths AND limitations associated with this case report	221-225
	11b	Discussion of the relevant medical literature **with references**.	213-219
	11c	The scientific rationale for any conclusions (including assessment of possible causes)	221-225
	11d	The primary "take-away" lessons of this case report (without references) in a one paragraph conclusion	236-240
Patient Perspective	12	The patient should share their perspective in one to two paragraphs on the treatment(s) they received	
Informed Consent	13	Did the patient give informed consent? Please provide if requested	Yes ☒ No ☐
